# Designing and Testing of Novel Taxanes to Probe the Highly Complex Mechanisms by Which Taxanes Bind to Microtubules and Cause Cytotoxicity to Cancer Cells

**DOI:** 10.1371/journal.pone.0129168

**Published:** 2015-06-08

**Authors:** Marc St. George, Ahmed T. Ayoub, Asok Banerjee, Cassandra D. M. Churchill, Philip Winter, Mariusz Klobukowski, Carol E. Cass, Richard F. Ludueña, Jack A. Tuszynski, Sambasivarao Damaraju

**Affiliations:** 1 Department of Laboratory Medicine and Pathology, University of Alberta, Edmonton, Alberta, Canada; 2 Department of Chemistry, University of Alberta, Edmonton, Alberta, Canada; 3 Department of Biochemistry, University of Texas Health Science Center, San Antonio, Texas, United States of America; 4 Department of Oncology, University of Alberta, Edmonton, Alberta, Canada; 5 Department of Physics, University of Alberta, Edmonton, Alberta, Canada; Taipei Medical University, TAIWAN

## Abstract

Our previous work identified an intermediate binding site for taxanes in the microtubule nanopore. The goal of this study was to test derivatives of paclitaxel designed to bind to this intermediate site differentially depending on the isotype of β-tubulin. Since β-tubulin isotypes have tissue-dependent expression—specifically, the βIII isotype is very abundant in aggressive tumors and much less common in normal tissues—this is expected to lead to tubulin targeted drugs that are more efficacious and have less side effects. Seven derivatives of paclitaxel were designed and four of these were amenable for synthesis in sufficient purity and yield for further testing in breast cancer model cell lines. None of the derivatives studied were superior to currently used taxanes, however computer simulations provided insights into the activity of the derivatives. Our results suggest that neither binding to the intermediate binding site nor the final binding site is sufficient to explain the activities of the derivative taxanes studied. These findings highlight the need to iteratively improve on the design of taxanes based on their activity in model systems. Knowledge gained on the ability of the engineered drugs to bind to targets and bring about activity in a predictable manner is a step towards personalizing therapies.

## Introduction

The taxanes, including paclitaxel and docetaxel, target tubulin, the subunit protein of microtubules, and bind to a well-characterized site on β-tubulin [1]. The mechanisms of binding and action, however, are highly complex. Unlike other anti-tubulin drugs, the taxanes specifically target the intact microtubule and their binding site is in the microtubule lumen [2]. Previous work by Freedman et al. [2] mapped the nanopores along the microtubule surface through which taxanes need to pass in order to reach the binding site. A specific site in the nanopore was identified as an intermediate taxane binding site. A second issue is that there are multiple isotypes of β-tubulin and that these differ both in their affinity for taxanes and their subcellular roles and locations [3]. Paclitaxel appears to exert its greatest effect on the βII isotype [4], which is also the major β-tubulin isotype in neurons [3], possibly accounting for the neuropathy associated with the taxanes. The βIII isotype, which is very abundant in aggressive tumors and much less common in normal tissues, would seem to be a good alternative target [5,6]. Our goal was to rationally design and test novel taxane derivatives that would bind to the intermediate binding site with differential affinity depending on the β-tubulin isotype expressed in cells.

Taxanes are among the most active antitumor agents in the treatment of various types of cancer, in particular breast, ovarian and lung cancer [7,8]. Resistance to taxanes is a problem for successful chemotherapy and can potentially arise from at least three distinct mechanisms. First, there is the classic mechanism arising from the action of P-glycoprotein (P-gp, encoded by the *MDR1* gene), which essentially pumps drugs out of the cancer cells [9]. Structurally different taxanes could, in principle, have different susceptibilities to the action of P-gp. Second, β-tubulin, the target of taxanes, exists as numerous isotypes differing in amino acid sequence and encoded by different genes [3]. One of the taxanes, paclitaxel, has its strongest effects on the βII isotype [4]. Since βII is over-expressed in many tumors [10] this is not surprising, however, βII is also a major component of the nervous system [5], which may account for the neurotoxicity of the taxanes. The βIII isotype would be a better target since it occurs largely in neurons but at much lower levels than βII, while its expression is very common in aggressive and metastatic cancers [6]. Third, the binding of taxanes to microtubules is very complex, and the drug has to traverse from the exterior milieu to the binding site in the lumen (interior) of the microtubule [11] to bring about the catalytic sequence of events leading to polymerization or depolymerization. This means that the taxane first has to bind to an intermediate site on the surface of the microtubule and then make its way inside. This intermediate site also differs among the isotypes. The drugs described here were designed and tested both *in vitro* and *in silico* in an effort to address all three of the above issues.

**Fig 1:**
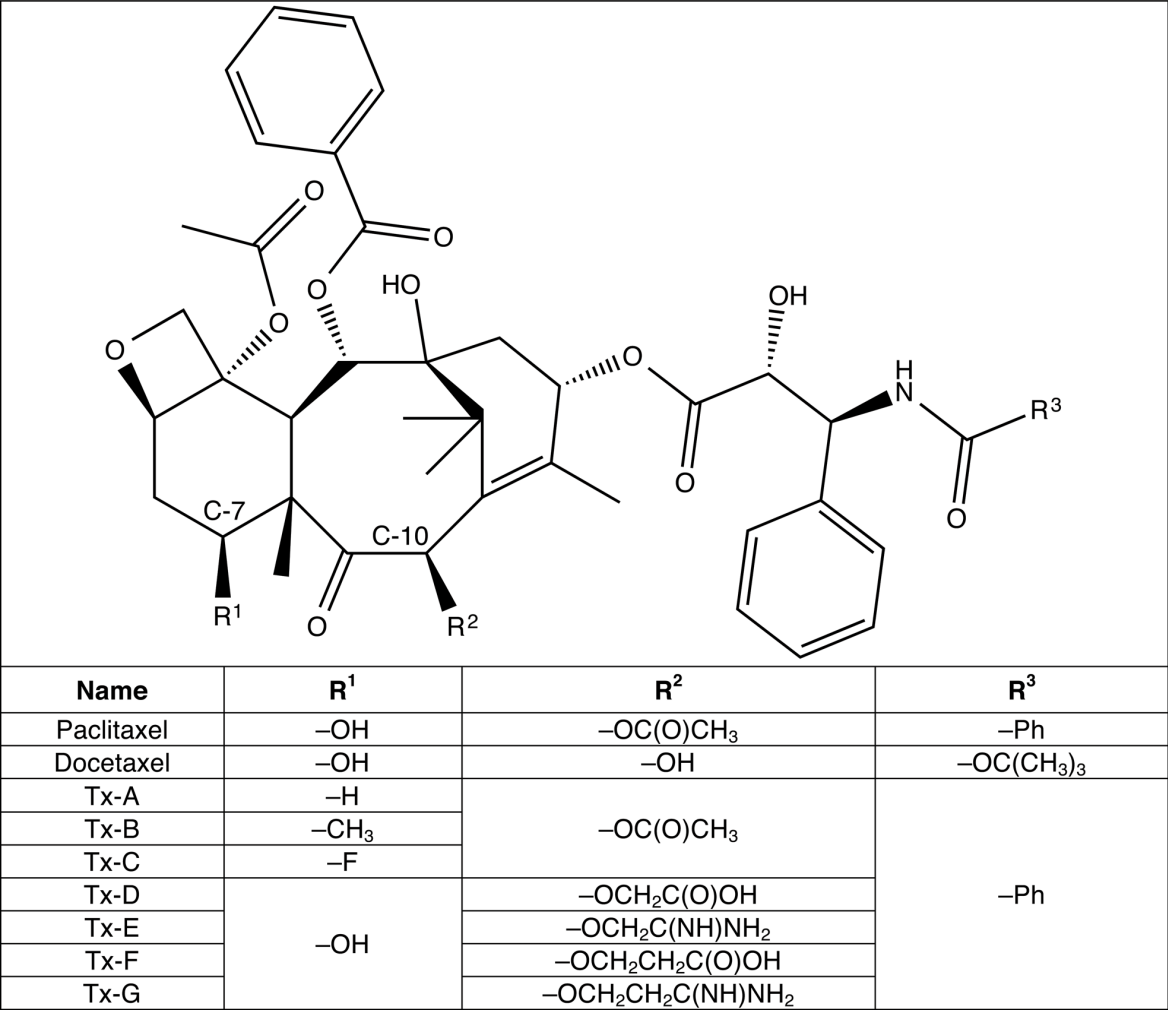
Chemical Structures of Paclitaxel, Docetaxel, and Paclitaxel Derivatives.

The chemical structures of paclitaxel and docetaxel are shown in Fig 1. The second-generation semi-synthetic drug docetaxel differs at two positions from paclitaxel. The substitution of the acetate ester at the C-10 position with a hydroxyl group makes docetaxel more water-soluble and bioavailable than paclitaxel [12,13], and as a result docetaxel is the favored of the two compounds for use in clinical applications. Many third-generation drugs are being developed to improve upon the parental compounds, with the goals of improving water solubility, tumor permeability, and cellular retention, thereby resulting in better outcomes and longer survival times for patients [14].

Taxanes specifically target the β-tubulin subunit of the α/β-tubulin heterodimer [15,16]. The location of the binding site is found on the lumen of the hollow microtubule structure. Given that microtubules are composed of repeating globular proteins, interstitial spaces (nanopores) are located along the length of the microtubule between adjacent protofilaments [2]. It is through these nanopores that taxanes have been shown to move and gain access to the binding site on the lumen [11,17]. Once bound to the final binding site a conformational change occurs, wherein a motif of β-tubulin known as the M-loop is stabilized, preventing the disassembly of the microtubules [16]. Taxanes bind in a 1:1 ratio with heterodimers along the length of the microtubule [18]. Although taxanes can potentially be bound to each heterodimer in a microtubule structure, only one taxane molecule is required to stabilize hundreds of tubulin heterodimers [18,19].

Both α-tubulin and β-tubulin are found in multiple isotypes expressed by different genes [20]. There are at least eight human β-tubulin isotypes, and the expression of these isotypes has been observed to differ in normal tissues and tumor samples [21]. Class III β-tubulin (βIII) in particular has been observed to be overexpressed in many forms of cancer, particularly drug-resistant cancer [6], whereas βI is constitutively expressed, and βII is highly expressed in brain tissue [21]. Based on consideration of the structure of microtubules, the intermediate binding site in the nanopore, and the sequence variations between β-tubulin isotypes, we hypothesized that taxanes engineered to interact differentially with tubulins will exert biological activities influenced by steric factors and/or affinities of engineered moieties to binding sites, relative abundance of tubulin isoforms, barriers imposed by lipid bilayers (relative solubility) and free energies of association or dissociation [2]. The specific objectives addressed in this study are:

- To investigate the hydrogen bonding between the C-7 hydroxyl of taxanes with Ser275 in *α*I and *β*II, their relative interactions with the nanopore, and the resulting diffusion of taxanes to the binding site.
- To investigate the hydrogen bonding between conserved Ser278 in tubulin isotypes (*β*I, *β*II and *β*III) with varying side chains engineered at the C-10 of taxanes, their relative interactions with the nanopore and the resulting diffusion of taxanes to the binding site.

The underlying premise for the first objective is based on computational modeling, which found that in βIII tubulin, which has alanine instead of serine at position 275, is unable to form a hydrogen bond with the C-7 hydroxyl in paclitaxel, which in turn weakens the interaction of paclitaxel with the nanopore, inhibits diffusion of the paclitaxel to the intermediate binding site, and results in an effective loss of paclitaxel activity in microtubules containing βIII tubulin [2].

The testing of the first objective requires the synthesis of paclitaxel derivatives with the C-7 hydroxyl replaced by a non-polar functional group (Tx-A and Tx-B, Fig 1) or fluorine (Tx-C, Fig 1). We expected to see decreased promotion of polymerization by paclitaxel for microtubules composed of the βIII isotype. When either Tx-A or Tx-B is tested however, we expect to see that the promotion of polymerization is less affected by the β-tubulin isotype; in other words, the effects of Tx-A or Tx-B should be insensitive (or at least less sensitive) to the β-tubulin isotype. Tx-C should have an intermediate activity, due to the lower energy associated with hydrogen bonding to the fluorine atom (3 kcal/mol compared to 5 kcal/mol). We were able to synthesize compounds Tx-A and Tx-C, and these were tested with *in vitro* polymerization assays or with the cell line models for cytotoxic activity.

The premise for the second objective is that a gap of between 4 and 9 Å needs to be spanned to access Ser278, and hence a side chain moiety could help bridge this gap. Hydrogen bonding between paclitaxel and Ser278 is predicted to cause the stabilization of the taxane interaction at the nanopore, resulting in faster movement of the drug to the binding site inside the microtubule, enhanced microtubule polymerization, and increased cytotoxicity. The testing of the second objective requires the synthesis of several modifications at the C-10 position of paclitaxel. We designed taxanes having different chain lengths on the C-10 position using either a guanidinium (compounds Tx-E and Tx-G, Fig 1) or a carboxylate (compounds Tx-D and Tx-F, Fig 1) functional group. These compounds are expected to be more active than paclitaxel. We were able to synthesize compounds Tx-D and Tx-F and these were tested with *in vitro* polymerization assays or with cell line models for cytotoxic activity.

## Materials and Methods

### Chemical Synthesis

We initially purchased compounds Tx-A, Tx-C, Tx-D and Tx-F (from among the *in silico* designed compounds Tx-A through Tx-G; Fig 1) due to their relative ease of synthesis in sufficient yield and relative purity to test the proposed objectives. The identities of the compounds were assessed by nuclear magnetic resonance (NMR), and purity by thin layer chromatography (TLC); compounds Tx-A, Tx-C, Tx-D and Tx-F were 88%, 94%, 94% and 96% pure, respectively, with yields of 6%, 10%, 3.6% and 3.3%, respectively. All compounds were custom synthesized through contract services provider Helios Biotech Ltd., Edmonton, Alberta, Canada.

### Cell Culture

Breast cancer cell lines SK-BR-3 [22], MDA-MB-231 [23], and T-47D [24] were kindly provided by Dr. Ing Swie Goping (University of Alberta, Canada). These are representative breast cancer cell lines reflecting the molecular heterogeneity observed in tumors in a clinical setting. The selected cell lines aid in the generalizability of findings and help to rule out a direct influence of the genotype-phenotype of a cell line on the binding and cytotoxic potential of paclitaxel and its analogs. Furthermore, the selected breast cancer cell lines have been previously reported to exhibit varying degrees of paclitaxel resistance [25], and our interest was to replicate these findings and to interrogate if the intrinsic resistance hampers the structure-activity relationships in taxane analogs described in the study objectives. Breast cancer tumors are characterized in terms of their expression of various cell surface receptors such as HER2 (human epidermal growth factor receptor 2), estrogen receptor (ER) and progesterone receptor (PR), which are involved in cell signaling and cellular proliferation.

- The SK-BR-3 cell line is HER2^+^, ER^−^ and PR^−^.
- The MDA-MB-231 cell line does not express or has very low expression for the receptors (HER, ER or PR) and is termed triple negative. Patients with triple negative receptor status undergo treatments with cytotoxic therapies such as paclitaxel or docetaxel.
- The T-47D cell line is ER^+^ but HER2^−^. Tumors that are ER^+^ and HER2^−^ (whether PR^+^ or PR^−^) are called luminal A tumors.

The prognosis is best for luminal A and worst for triple negative; HER2^+^ tumors (Her2^+^ and ER^−^) have an intermediate prognosis [26].

A second set of non-breast cancer cell lines were also used to assess the generality of findings with regard to paclitaxel and its analogs, except that these cell lines are well characterized for P-glycoprotein (P-gp) expression, and are classified as either drug sensitive or resistant phenotypes. MES-SA (P-gp negative, wild type) [27] and MES-SA/Dx5 (P-gp positive, drug resistant) [28] uterine sarcoma cell lines, and K-562 (P-gp negative, wild type) [29] and K-562/R7 (P-gp positive, drug resistant) [30] chronic myelogenous leukemia cell lines were kindly provided by Dr. Charles Dumontet (University of Lyon, France). This panel was selected to determine if the changes made to the taxane structures had an effect on substrate specificity for the multidrug resistance efflux pump, P-gp. MES-SA and MES-SA/Dx5 are adherent cells, while K-562 and K-562/R7 are suspension cells. It has been previously reported that the MES-SA/Dx5 and K-562/R7 cell lines overexpress P-gp protein and exhibit cross-resistance to a variety of chemotherapeutic compounds [28,31].

All cell lines were maintained in Roswell Park Memorial Institute (RPMI) 1640 media supplemented with 10% fetal bovine serum (FBS), at 37°C, 5% CO_2_ and in a humidified environment. Adherent cells were maintained in media until ~90% confluent. Cells were trypsinized in a solution of Trypsin-EDTA (Gibco, Life Technologies Corporation, Carlsbad, CA, USA) at a final concentration of 0.25%.

### Microtubule Polymerization Assay

Bovine brain tubulin preparation and purification of the αβII and αβIII dimers (α-tubulin was a mixture of isotypes; only β-tubulin was purified into βII and βIII, respectively) were performed as previously described [32]. Microtubule polymerization assays were performed as previously described [33]. Aliquots (200 μl) of isotypically pure bovine brain tubulin (1.4 mg/ml) in tubulin buffer (100 mM MES-Na, 1 mM EGTA, 0.1 mM EDTA, 0.5 mM MgCl_2_, 1 mM GTP, pH 6.4) were incubated in the presence of drugs (1–16 μM) at 37°C for 15 min in a DU model spectrophotometer (Beckman Coulter, Inc., Indianapolis, IN, USA) and the turbidity of the samples were monitored at 350 nm every 8 s. The raw data were acquired and processed using KINLOTUS software. The raw data were subsequently baseline-corrected and the turbidity values were plotted against time.

### MTS Assay

Cell viability in the presence of drug was determined using the CellTiter 96 AQ_ueous_ Non-Radioactive Cell Proliferation Assay (MTS) (Promega, Madison, WI, USA). Cells were seeded in 96-well plates (Nalge Nunc International, Rochester, NY, USA) at levels proportional to their growth rate (ranging from 5 to 10 thousand cells per well) at a volume of 100 μl and allowed to adhere to the plate overnight. The following day the cells were treated with a range of 100 μl of 2x drug concentrations for a final 1x drug concentration per well. Each drug concentration was administered to six different wells, to assess technical reproducibility of the assay. This treatment was maintained unchanged for a 72 hour period. Following 72 hours of treatment, 30 μl of MTS reagent was added to each well. The plates were incubated at 37°C for a time period of 30 minutes to 4 hours, depending on color development, a rate that was variable from cell line to cell line. Relative cell viability was determined by measuring the absorbance of each well at 490 nm and converted to a percent of total viability compared to an untreated control. Experimental results are an average of at least n = 3 experiments.

### SDS-PAGE

Sodium Dodecyl Sulfate Polyacrylamide Gel Electrophoresis (SDS-PAGE) analysis was performed for western blotting. 10% polyacrylamide gels (from a stock solution of 40% 29:1 acrylamide/bis solution, 0.375 M Tris, 0.1% (v/v) SDS, 0.1% (v/v) ammonium persulfate, 0.1% (v/v) TEMED (N,N,N',N'-Tetramethylethylenediamine), water to volume) were used for all western blot experiments. Polyacrylamide (7.5%) gels were used for western blot analysis of P-gp protein (170 kDa) for the required resolution of protein bands in this size range. Total protein lysates (25 μg) were loaded into 10 or 15 well polyacrylamide gels using 1x Laemmli Sample Buffer (Bio-Rad Laboratories Inc., Hercules, CA, USA). Gels were run at 200 V for ~1 h in 1x Tris/Glycine/SDS run buffer (ICN Biomedicals Inc., Irvine, CA, USA) before being removed and stained with Coomassie Blue or transferred to nitrocellulose membrane for western blot analysis. Coomassie-stained gels were destained using a solution of 50% H_2_O, 40% methanol, and 10% glacial acetic acid. Destained gels were scanned on a CanoScan LiDE 600F (Canon Inc., Tokyo, Japan) and visualized using Adobe Photoshop 6.0 software (Adobe Systems, San Jose, CA, USA).

### Western Blot Analysis

Following gel electrophoresis and transfer of proteins to nitrocellulose membranes, the membranes were blocked with 5% Tris buffered saline (TBS), plus milk and Tween (TBSMT) (154 mM Tris HCl, 1.37 M NaCl, 0.1% (v/v) Tween20, 5% (w/v) milk) solution for 1 hour. Proteins on membranes were exposed overnight to primary antibody-TBSMT solution. On the following day, membranes were washed in Tris buffered saline, plus Tween (TBST) (154 mM Tris HCl, 1.37 M NaCl, 0.1% (v/v) Tween20, pH 7.6), for 6 cycles with 5 min wash/incubation for each cycle in TBST to remove unbound antibody. Membranes were further exposed to secondary antibody in TBST for 30 min. Following secondary antibody incubation, membranes were washed in TBST for 12 cycles with 5 min wash/incubation for each cycle in TBST to remove any unbound secondary antibody. Proteins were detected following a 5 min exposure to either ECL Prime Western Blotting Detection Reagent (GE Healthcare, Little Chalfont, England) or Lumi-Light Western Blotting Substrate (Roche Diagnostics, Indianapolis, IN, USA) and the exposed film was developed using a KODAK m35A X-OMAT Processor (Eastman Kodak, Rochester, NY, USA). Proteins detected using primary mouse anti-human antibodies were: βIII (ab14545; Abcam, Cambridge, England) used at a dilution of 1 in 1000, P-gp (ab3366; Abcam, Cambridge, England) used at a dilution of 1 in 2000, and goat anti-human actin (sc-1616; Santa Cruz Biotechnology Inc., Dallas, TX, USA) used at a dilution of 1 in 4000. Secondary antibodies were goat anti-mouse (115-035-003; Jackson ImmunoResearch Laboratories Inc., West Grove, PA, USA), at a dilution of 1 in 10,000 and rabbit anti-goat (305-035-045; Jackson ImmunoResearch Laboratories Inc., West Grove, PA, USA) used at a dilution of 1 in 40,000. The original films were scanned and the images were imported into PowerPoint. The bands located at the expected size range were cropped out of the films. Size and contrast of each figure was adjusted such that all figures matched in size and then grouped together. The contrast of the entire group was adjusted so that bands could be better visualized and the color was consistent.

### Statistical Analysis

GraphPad Prism 6 (GraphPad Software, Inc., La Jolla, CA, USA) was used to create figures and perform statistical analyses of results. Standard Student’s T-test was used to compare the cytotoxicity results for each of the analogs relative to paclitaxel induced cytotoxic profiles; as also for all cytotoxic assay results with and without other variables (e.g., verapamil). One way ANOVA (analysis of variance) was used to show the statistical significance between low, intermediate and high drug resistance in cell lines treated with paclitaxel and derivatives.

### Computational Simulations

#### Binding to the Taxane Binding Site

The coordinates of the tubulin-taxane receptor-ligand complex were obtained from the PDB crystal structure 1JFF [34], which represents paclitaxel co-crystallized with tubulin. The coordinates of this crystal structure were used to build the complexes of the other taxane derivatives. The structures were built using Avogadro 1.0.3 software [35]. Nine complexes were built, including Tx-A through Tx-G as well as paclitaxel and docetaxel as controls. The receptor was prepared by removing the alpha subunit of 1JFF, as it does not directly contribute to the binding of taxanes. The missing first methionine residue was added and the C-terminus was capped with an N-methyl residue. The cofactor guanosine diphosphate (GDP) was also included in the structure and its parameters were obtained from Meagher et al. [36] Ionization states of protein residues were assigned using the PROPKA server [37–39]. The ligands were parameterized according to the general AMBER force field (GAFF) [40] and partial charges were assigned with the AM1-BCC method [41] using the *antechamber* module of AMBER 12 [42]. The complexes were then built using the *tleap* module of AMBER 12 in which the protein was parameterized using the AMBERff12SB force field [43,44]. The complex was solvated in a truncated octahedral box extending 12 Å in each direction. The complex was then neutralized by the addition of 16 sodium ions. Another 22 sodium and chloride ions were added to achieve a salt concentration of 100 mM. Each complex was then minimized with heavy restraints (500 kcal/(mol Å)) on the protein and without SHAKE on hydrogen atoms for 1000 steepest descent runs followed by 1000 conjugate gradient runs. Another minimization with 3000 steepest descent runs followed by 3000 conjugate gradient runs was performed without any restraints. Heating to a temperature of 300 K under constant volume with SHAKE and restraints on the protein was performed using a Langevin thermostat for 20 ps. Density equilibration at constant pressure for 200 ps was then performed using SHAKE on hydrogen atoms and under gradually decreasing restraints on heavy atoms. This was followed by a production phase of 10 ns at 300 K under constant pressure where coordinates were recorded every 2 ps. All the simulations were done under periodic boundary conditions using the particle mesh Ewald method for the treatment of long-range electrostatics. Density, temperature, total energy and RMSD were checked for equilibration. The last 2 ns of the production run were post-processed for the binding free energy calculation using the Molecular Mechanics/Poisson–Boltzmann Surface Area (MM/PBSA) [45] or Molecular Mechanics/Generalized Born Surface Area (MM/GBSA) approaches [46]. We extracted 200 evenly-spaced snapshots from the last 2 ns of the production run and used them for the MM/PBSA and MM/GBSA calculations. We also performed normal mode analysis for each complex using 100 evenly-spaced snapshots which were extracted from the last 2 ns of the production run. Since normal mode analysis is very time-consuming, we used an approach in which the protein is truncated. Specifically, all residues farther than 12 Å from the ligand were truncated, and the analysis was performed only on the remaining system. This approach has been proven efficient and sufficiently accurate by Genheden et al [46].

In our approach, the binding free energy is calculated according to the formula:

$$\bar{G}={\bar{E}}_{MM}+{\bar{G}}_{PB(GB)SA}-TS_{MM},$$ (1)

where ${\bar{E}}_{MM}$ is the average molecular mechanical energy including contributions from bond, angle, dihedral, van der Waals and electrostatic terms. ${\bar{G}}_{PB(GB)SA}$ is the solvation free energy calculated by solving the Poisson–Boltzmann (or Generalized Born) equation to obtain the polar solvation free energy and by estimating non-polar solvation free energy using a surface area term. −*TS*_*MM*_ is the entropy term estimated using normal mode analysis. Once the average free energy is calculated for the ligand, receptor, and complex, the binding energy is obtained through the following equation:

$$\Delta G={\bar{G}}_{\text{complex}}-{\bar{G}}_{\text{receptor}}-{\bar{G}}_{\text{ligand}}.$$ (2)

#### Binding to the Intermediate Nanopore Site

A microtubule nanopore model was constructed by combining electron microscopy data for a microtubule (1XRP) [47] with X-ray data for an αβ-tubulin heterodimer (1JFF) [34]. Beginning from the protein and nucleotide components of the 1JFF crystal, missing residues (specifically α:1,35–60 and β:1) were taken from 1TUB [1] and overlaid onto 1JFF. Next, the protonation state of ionizable residues in 1JFF was determined using PROPKA and hydrogen atoms added to obtain a complete αβ-tubulin heterodimer. Finally, this dimer was used to construct a portion of a microtubule using electron microscopy data from 1XRP. The model used was for the type 1 pore [2], and only the four tubulin monomers adjacent to the pore were retained.

**Fig 2:**
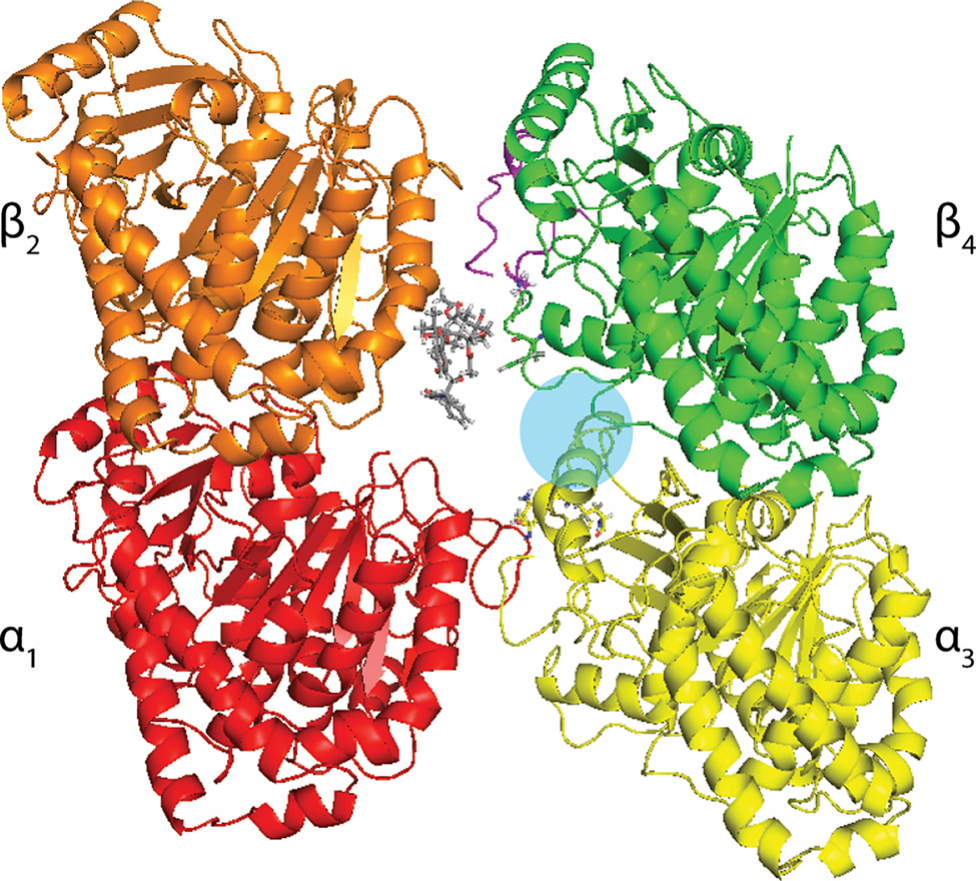
Computational Model Used to Describe Paclitaxel Interactions in the Intermediate Binding Site Located in the Type 1 Microtubule Nanopore. Nanopore is viewed from microtubule exterior. The blue circle indicates the intermediate binding site.

The binding mode of paclitaxel to the intermediate binding site was taken from Freedman et al. [2] and overlaid onto the nanopore structure described above, resulting in the nanopore-paclitaxel model used in this study (Fig 2) as a starting structure to define the intermediate binding site in docking calculations. Note that interdimer contacts have not been equilibrated in this model. The binding mode of paclitaxel in the intermediate binding site reported by Freedman et al. differs from that reported by Maccari et al. [48], which is located closer to α_3_-tubulin. It is likely that both sites are stable states along the same internalization pathway.

Docking calculations were performed with the Molecular Operating Environment (MOE; Chemical Computing Group, Montreal, Canada). MOE was selected for docking due to its induced-fit docking protocol and ability to easily model ligands with different charges and ionization states. The four heterodimers composing the type 1 pore were used to define the receptor, and five taxanes (paclitaxel, Tx-A, Tx-C, Tx-D and Tx-F) were docked to the site identified by Freedman et al. [2]. Due to the low pKa of the carboxylic acid functional groups in Tx-D and Tx-F, these two taxanes were modeled as anionic (deprotonated) to best represent their structure at a physiological pH. Scoring was initially calculated with the London dG scoring function, retaining 30 conformers (with duplicates removed). Subsequently, refinement was performed using the Forcefield method and additional rescoring using the GBVI/WSA dG scoring function. Four different docking protocols were used with varying sizes of binding site and with either a flexible or a rigid receptor.

## Results and Discussion

### Tubulin Polymerization Using Paclitaxel and Synthesized Derivatives

Tubulin polymerization assays were performed using the paclitaxel derivatives to measure *in vitro* polymerization activity compared to paclitaxel. These experiments used affinity-purified βII and βIII tubulin isotypes from a bovine brain source; the α-tubulin was a mixture of isotypes; these forms of tubulin are referred to as αβII and αβIII, respectively.

**Fig 3:**
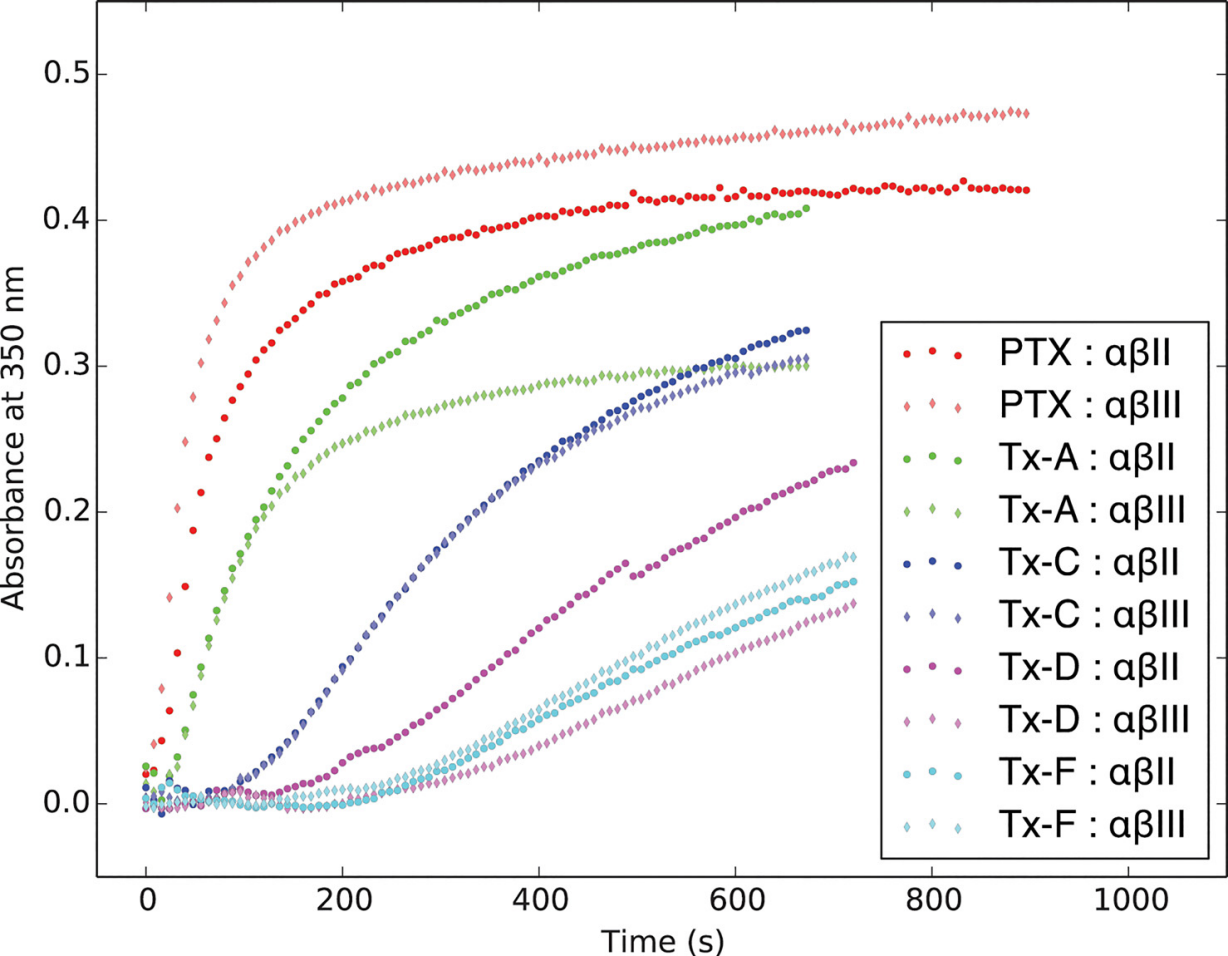
Microtubule Polymerization in the Presence of Paclitaxel and Derivatives with αβII and αβIII. Isotypically pure bovine brain tubulin (αβII or αβIII) at 1.4 mg/ml was incubated in the presence of drugs and the absorbance at 350 nm was measured every 8 seconds for at least 11 minutes. The concentration of each drug was 10 μM. PTX is paclitaxel.

Polymerization curves were established using paclitaxel and four of its derivatives with either αβII and αβIII. The polymerization curves at drug concentrations of 10 μM are shown in Fig 3A drug concentration of 10 μM was the concentration at which the differences between the effects of all the drugs were most clearly visible; however the same trends were observed at all concentration levels tested (1–16 μM).

The results show that paclitaxel corresponds to the highest rate of microtubule assembly. This is followed by Tx-A, then by Tx-C. Tx-D and Tx-F had similar values, with the slowest rates of microtubule assembly. None of the derivatives tested surpassed paclitaxel in terms of the rate of tubulin polymerization. There was an increase in the assembly rate for paclitaxel with followed by Tx-A, then by Tx-C. Tx-D and Tx-F had similar values, with the slowest rates of microtubule assembly.

The results were not consistent with earlier predictions [2] and the underlying premise described for the first objective: paclitaxel was not observed to have decreased polymerization with βIII tubulin (the observation was the reverse of the expectation), and furthermore Tx-A and Tx-C did not have the expected behavior. Neither were the results consistent with the underlying premise described for the second objective: Tx-D and Tx-F were not more active than paclitaxel. Therefore, this experiment does not support the importance of residues 275 or 278, or the predicted role of the intermediate binding site in the microtubule nanopore.

#### Cytotoxicity of Paclitaxel Analogs Against Breast Cancer Cell Lines

**Fig 4:**
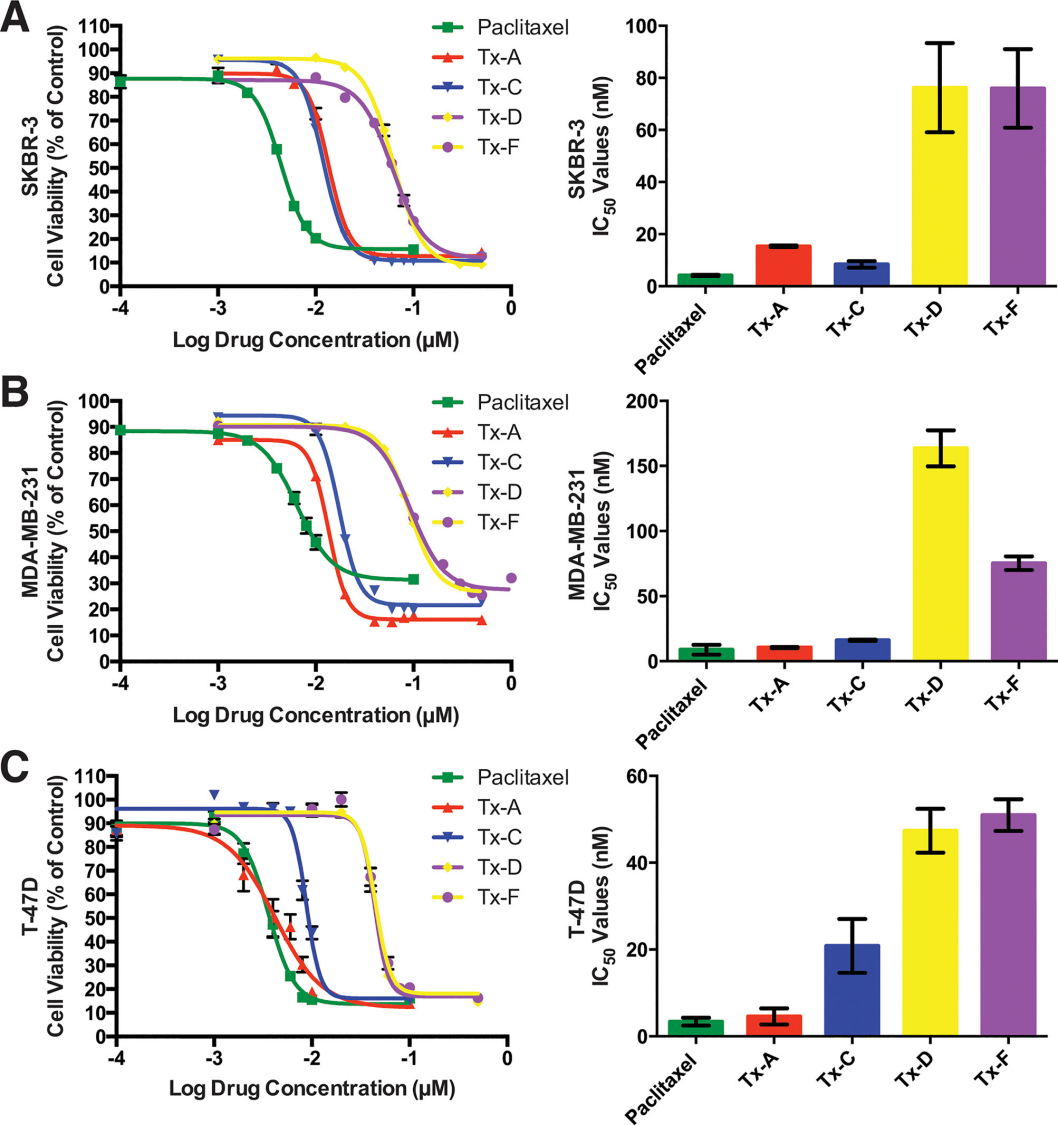
IC_50_ Values for Paclitaxel and Analogs in Cytotoxicity Assays with Breast Cancer Cell Lines. Cell lines were treated with a range of drug concentrations as indicated (left plot in each panel) to assess the cytotoxic activity of paclitaxel and analogs. Cell lines were exposed to drugs for 72 h. 30 μl of MTS reagent was administered to each well, and absorbance measurements were taken at 490 nm. Cell lines studied were: (A) SK-BR-3 (HER2^+^), (B) MDA-MB-231 (triple negative) and (C) T-47D (luminal A). All values are averages of replicates expressed relative to cell viability values in untreated cells normalized to 100%. Cytotoxicity curves represent n = 3 experiments with 6 replicates per drug concentration for each experiment. Bar graphs (right plots in each panel) show the IC_50_ value of n = 3 independent experiments. Standard deviations, SD, are shown for each drug concentration (left plots) and for each IC_50_ determined (right plots). * indicates P < 0.05 determined for IC_50_ for an analog relative to paclitaxel.

Cell line experiments were carried out to further understand the findings obtained from the experiments with purified protein. Cells were treated with either paclitaxel or one of the analogs at a range of concentrations for 72 h. The MTS assay was used to determine relative cell viability at each drug concentration compared to the untreated control (Fig 4). The data from the drug titration curves were analyzed for IC_50_ (the concentration where 50% of the effect on cell viability was observed; lower IC_50_ values correspond to drugs with more potency; Table 1, Fig 4).

**Table 1:** Summary of Drug Titrations Showing 50% Inhibitory Concentration, IC_50_, in Breast Cancer Cell Lines Treated with Paclitaxel and Analogs.

	Paclitaxel	Tx-A	Tx-C	Tx-D	Tx-F
SK-BR-3	5±1	16±1*	9±2*	77±18*	76±16*
MDA-MB-231	9±4	11±1	17±1	164±14*	76±6*
T-47D	4±1	5±2	21±6*	48±6*	51±4*

The IC_50_ values are given in nM. Values and errors (SE) shown are representative of n = 3 independent experiments.

* P < 0.05 determined for IC_50_ for each analog relative to paclitaxel.

#### Drug titrations with SK-BR-3

The dose response curves and IC_50_ values from the MTS assay are shown in Fig 4A. The rank order for the drugs from most potent to least potent was paclitaxel > Tx-C > Tx-A > Tx-F ≈ Tx-D based on the IC_50_ values. All four derivatives showed statistically significant differences in IC_50_ compared to paclitaxel.

#### Drug titrations with MDA-MB-231

This cell line was the most resistant to paclitaxel treatment of the three cell lines tested, with double the IC_50_ value for paclitaxel compared to other two cell lines. Dose response curves and IC_50_ values are shown in Fig 4B. The rank order of potency was paclitaxel ≈ Tx-A > Tx-C > Tx-F > Tx-D. Only Tx-D and Tx-F showed statistically significant differences in IC_50_ values compared to paclitaxel.

#### Drug titrations with T-47D

This cell line had a similar IC_50_ value for paclitaxel compared to the SK-BR-3 cell line. The dose response curves and IC_50_ values are shown in Fig 4C. The rank order of potency was paclitaxel ≈ Tx-A > Tx-C > Tx-D ≈ Tx-F. The derivatives Tx-C, Tx-D and Tx-F showed statistically significant differences compared to paclitaxel.

The overall trend for all three cell lines was that paclitaxel was the most potent, with Tx-A having potency very close to paclitaxel or slightly less, Tx-C with intermediate potency, and with Tx-D and Tx-F being the least potent. Even though no drugs showed improvement in IC_50_ values relative to the parent compound, the IC_50_ values were in the nM range (Table 1), therefore the analogs are still considered to be cytotoxic. MDA-MB-231, which does not express cell surface receptors (ER, PR and HER2) was the most resistant to paclitaxel; this compares well with the observations in the clinic that triple negative breast cancers often show mutations that confer intrinsic resistance to chemotherapeutic agents. There were no clear differential activities against the three cell lines that would suggest that any of the derivatives should be preferred over paclitaxel for tumors with the three different receptor expression profiles.

The data obtained from the microtubule polymerization assays and cell line cytotoxicity assays showed agreement. Paclitaxel was both the strongest inhibitor of polymerization and the most cytotoxic compound. Tx-A had the closest effect to paclitaxel in the polymerization assay, and was also the closest to paclitaxel in the cytotoxicity assays. Tx-C had the next strongest effect on polymerization, and was the next most cytotoxic. Finally, Tx-D and Tx-F were both the weakest inhibitors of polymerization and the least cytotoxic compounds.

Examining the original premise regarding the effect of the modifications to paclitaxel on the activity of the taxane derivatives, only the case of Tx-C showed support. In this case we consistently observed a small but significant decrease in activity compared to paclitaxel, as originally expected. However, in the cases of the other three derivatives tested, the predicted patterns were not observed. In the case of Tx-A, there was either a slight decrease in activity, or even an increase in activity, whereas the prediction was that Tx-A should have a decrease in activity compared with paclitaxel. Finally, the derivatives Tx-D and Tx-F were expected to show increased activity relative to paclitaxel, however these two derivatives consistently had decreased activity.

#### Expression of the βIII Tubulin Isotype in Breast Cancer Cell Lines and its Association with the Observed Sensitivity of Cell Lines to Paclitaxel and Analogs

Western blot analysis was performed to investigate the role of βIII tubulin expression on the different cytotoxic effects of paclitaxel and analogs against the breast cancer cell lines. It was expected that differences in βIII tubulin expression will be correlated with the cytotoxicity of paclitaxel and the novel analogs, with higher βIII tubulin expression corresponding to more resistance to paclitaxel treatment. Furthermore, it was expected that taxanes Tx-A and Tx-C will have decreased cytotoxicity against cell lines that express βIII tubulin. The experimental protocol was also designed to determine if βIII expression of the cell lines under normal growth conditions is different from expression after exposure to paclitaxel. MDA-MB-231, SK-BR-3 and T-47D cells were grown in two distinct experimental groups. In the first experimental group, cells were grown in drug free media for 7 days, at which time cells were harvested and protein extracts were prepared for western blots. This experimental group was designed to measure the constitutive expression of βIII tubulin in the cell lines. In the second experimental group, the cells were treated similarly to those in the first group, except that paclitaxel was added after 6 days and they were incubated with the drug for the final 24 hours. The paclitaxel concentration used was one-half the previously determined IC_50_ for paclitaxel against each specific cell line (Table 1) so as to ensure that the drug was not administered at a concentration that would kill most of the cells, but would still be high enough to have an effect.

**Fig 5:**
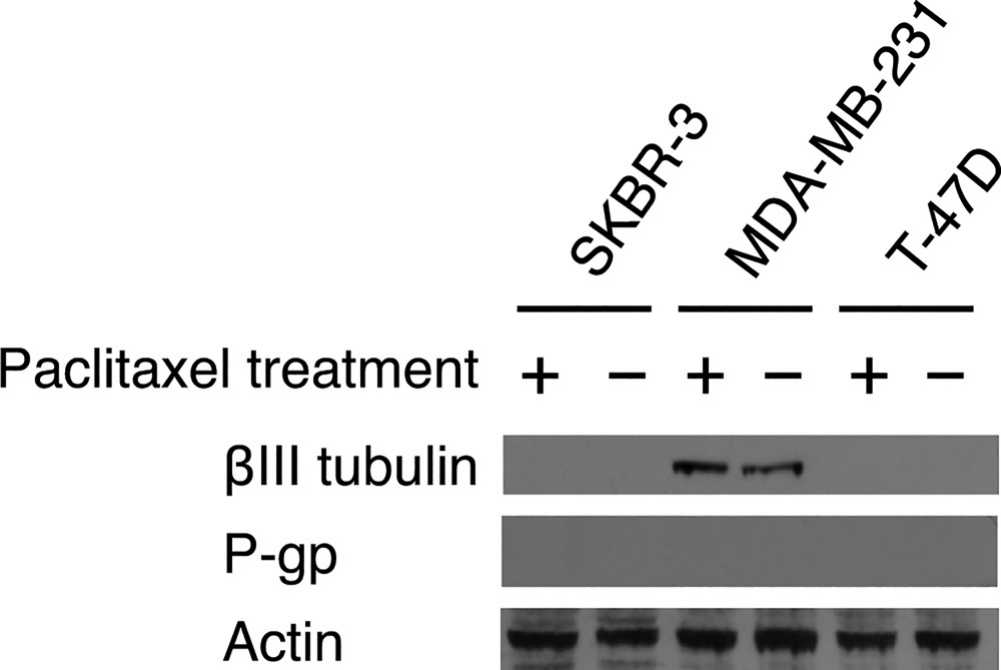
Western Blot Analysis of Expression of β-Tubulin Isotypes and P-gp for Breast Cancer Cell Lines. Breast cancer cell lines were used to determine expression of the βIII tubulin isotype and the P-gp (MDR1) efflux pump. Experiments were carried out under normal media conditions (−) or after 24 h paclitaxel exposure with concentration at one-half IC_50_ (+). The image is representative of the results from n = 3 independent experiments. Actin expression on the blot was used as a loading control.

The data showed that there was variability in the βIII tubulin isotype expression between the cell lines: MDA-MB-231 was the only cell line of the three to show any expression of βIII tubulin (Fig 5). MDA-MB-231 was also the most resistant of three cell lines to paclitaxel (Table 1). Therefore, these results are consistent with the expectation that increased βIII tubulin expression is correlated with increased resistance to paclitaxel. However, there was no correlation between βIII tubulin expression and the cytotoxicity of either Tx-A or Tx-C, therefore the expectation that βIII tubulin expression would be correlated with decreased cytotoxicity of these two derivatives is not supported.

The results also showed that exposure to paclitaxel for 24 hours had no measurable effect on βIII tubulin expression in any of the three cell lines under these experimental conditions. Exposure to paclitaxel did not induce βIII tubulin expression in the cell lines that do not express it under untreated conditions, nor did it alter expression in the cell line (MDA-MB-231) with constitutive βIII tubulin expression.

#### Substrate Specificity of Paclitaxel Derivatives for Drug Efflux Pump P-gp

Paclitaxel is known to be a substrate of P-gp [9]. We investigated the effect of P-gp on paclitaxel resistance and on the novel analogs, which are also expected to be substrates of P-gp. Western blot analysis was used to determine the expression levels of P-gp for the panel of breast cancer cell lines used for the cytotoxicity experiment (Fig 5). The results showed that none of the three cells express any measurable level of P-gp, whether untreated or after exposure to paclitaxel for 24 hours. Therefore P-gp efflux does not appear to be the mechanism of resistance in these cell lines at the indicated time points used for the assays. Therefore a further series of experiments using different cell lines were designed specifically to test P-gp efflux of the taxanes, as described in the following section.

#### Cytotoxicity of P-gp Cell Lines

The cytotoxicity results for the second panel of cell lines (Table 2) provides additional information regarding the efficacy of the paclitaxel derivatives. The overall trend for the cytotoxicities of the analogs was similar to the trend for the panel of breast cancer cell lines. Tx-A had the potency closest to paclitaxel, and in one case even more potency: with the K-562/R7 cell line the IC_50_ value for Tx-A treatment was significantly improved over that of paclitaxel–this is the only example among the cases tested where a derivative was observed to have a statistically significant improvement over paclitaxel. Notably, K-562/R7 was also the cell line that was most resistant to paclitaxel among all the cell lines tested.

**Table 2:** Summary of Drug Titrations Showing 50% Inhibitory Concentration, IC_50_, for the P-gp^±^ Cell Lines Treated with Paclitaxel and Derivatives (and ± Verapamil).

	Paclitaxel	Tx-A	Tx-C	Tx-D	Tx-F
MES-SA	21±3	18±3	41±4*	289± 31*	314±69*
MES-SA + VRP^§^	13±1	20±5	39±4*	183±13*	138±11*
MES-SA/Dx5^¶^	18±4	134±33*	666±229*	1963±541*	1475±324*
MES-SA/Dx5^¶^ + VRP^§^	11±5	15±2	38±3*	288±24*	253±58*
K-562	6±2	9±2	57± 2*	669±69*	593±24*
K-562 + VRP^§^	5±2	6±1	10±1*	23±1*	24±2*
K-562/R7^¶^	440±26	266±27^‡^	1482±33*	5474±81*	4586±75*
K-562/R7^¶^ + VRP^§^	41±7	22±2	83±7*	552±5*	467±72*

The IC_50_ values are given in nM. Values and errors (SE) shown are representative of n = 3 independent experiments.

* P < 0.05 determined for IC_50_ for an analog relative to paclitaxel.

^‡^ P < 0.05 determined for IC_50_ for an analog with improved potency relative paclitaxel.

^§^ VRP is verapamil.

^¶^ P-gp^+^ cell line.

The cytotoxicity data (Table 2) show that there is generally a large increase in the IC_50_ values (decrease in potency) for the cell lines that express P-gp compared with the paired cell line that does not express P-gp. MES-SA and MES-SA/Dx5 with paclitaxel was an exception. To further confirm that the change in IC_50_ is due to P-gp efflux, cytotoxicity experiments were also performed with the addition of verapamil (VRP), a P-gp inhibitor (Table 2). The results support the statement that paclitaxel and its analogs are substrates for P-gp, as expected.

#### Expression of the βIII Tubulin Isotype in P-gp^±^ Cell Lines and Association with Observed Sensitivity of Cell Lines to Paclitaxel and Derivatives

**Fig 6:**
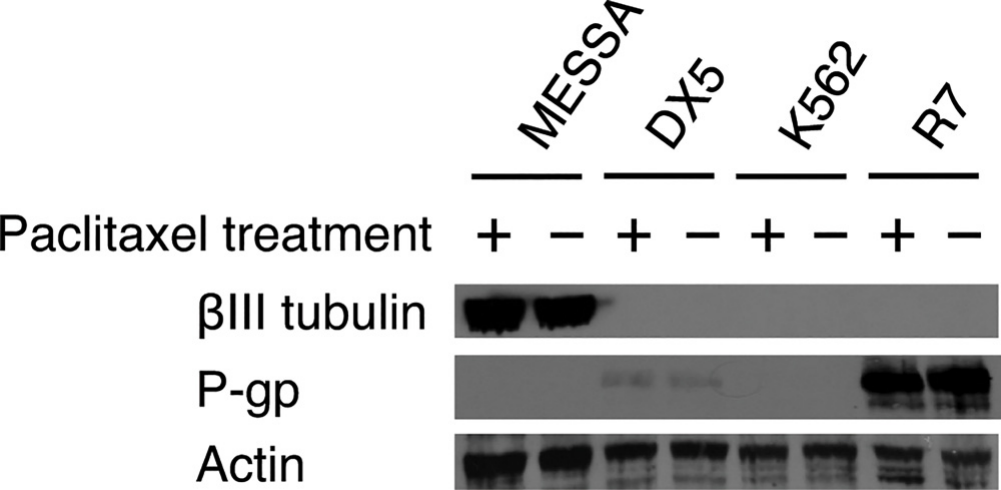
Western Blot Analysis of Expression of β-Tubulin Isotypes and P-gp for the P-gp^±^ Cell Lines. Two pairs of cell lines were used to determine expression of βIII tubulin isotype and P-gp efflux pump. Parental cell lines MES-SA (uterine sarcoma) and K-562 (chronic myelogenous leukemia) are P-gp negative, whereas daughter cell lines MES-SA/Dx5 and K-562/R7 express P-gp, and have been characterized in the literature as multidrug-resistant lines [28,30]. Experiments were carried out under normal media conditions (−) and after 24 h paclitaxel exposure with concentration at one-half IC_50_ (+). The image is representative of the results from n = 3 independent experiments. Actin protein was used as a loading control.

Western blot experiments were carried out using the second panel of cell lines to determine the levels of βIII tubulin expression (Fig 6). βIII tubulin expression was only observed in the MES-SA cell line, but not any other cell lines, including the MES-SA derivative, MES-SA/Dx5. The observed high expression of βIII tubulin may contribute to the equally strong drug resistance seen in both the MES-SA and MES-SA/Dx5 cell lines, with the resistance facilitated by different mechanisms; βIII expression in the case of MES-SA and P-gp expression in the case of MES-SA/Dx5. As with the breast cancer cell line panel, paclitaxel treatment had no effect on βIII tubulin expression.

#### Substrate Specificity of Paclitaxel Derivatives for Drug Efflux Pump P-gp

Using western blot analysis, we verified that both the MES-SA/Dx5 and K-562/R7 cell lines express P-gp, as expected, whereas MES-SA and K-562 showed no P-gp expression (Fig 6). MES-SA/Dx5 showed low P-gp expression, while K-562/R7 had high P-gp expression. Exposure of the cell lines to paclitaxel did not alter P-gp expression, as with the breast cancer cell lines.

The only case where the cytotoxicity results showed that there was no increase in IC_50_ value for a P-gp positive cell line is in the case of MES-SA/Dx5 treated with paclitaxel (Table 2). Since the Western blot showed that MES-SA/Dx5 cells have only a relatively low level of P-gp, a potential explanation for the cytotoxicity results is that the P-gp expression in MES-SA/Dx5 is not large enough to affect the potency of paclitaxel. We have not tested if prolonged growth or incubations of cell lines for up to 48 or 72 hours may effect expression of P-gp in MES-SA/Dx5 cell line. Nevertheless, it is reasonable to conclude that insensitivity to verapamil in MES-SA is expected, since an independent mechanism conferring relative resistance was observed, i.e. the higher expression of βIII tubulin. Furthermore, since the MES-SA/Dx5 cell line does have increased resistance to the taxane analogs (unlike with the parent paclitaxel), this suggests that the taxane analogs are even more susceptible to P-gp efflux than is paclitaxel.

#### Combination Treatments Containing Verapamil and Paclitaxel Analogs Contribute to Enhanced Cell Kill

The results are consistent with the predictions that the paclitaxel analogs are indeed substrates for P-gp (Table 2). We wished to further determine if drug resistance to paclitaxel analogs in two cell lines expressing P-gp, MES-SA/Dx5 and K-562/R7, can be reversed using the known P-gp inhibitor, verapamil [49]. Verapamil was used at a concentration of 5 μg/ml in combination with different concentrations of either paclitaxel or analogs to determine the IC_50_ values under these new conditions. The results were then compared to assays in which verapamil was absent. This analysis showed that paclitaxel and the analogs were more cytotoxic in the presence of verapamil when administered to MES-SA/Dx5 and K-562/R7 cells. In all experimental groups verapamil lowered the IC_50_ of the P-gp^+^ cell lines to near wild-type levels (Table 2). (There was no significant change in IC_50_ for the MES-SA/Dx5 cells treated with verapamil; however, as noted previously, the MES-SA/Dx5 cells did not show increased resistance to paclitaxel compared with parental MES-SA cells.)

Comparisons were made between parental and P-gp^+^ cell lines. MES-SA (P-gp^−^) and MES-SA/Dx5 (P-gp^+^) cells with and without verapamil treatment have statistically equal resistance to paclitaxel. Given that in MES-SA/Dx5 expression of P-gp is a mechanism of paclitaxel resistance, and MES-SA cells have an intrinsic resistance mechanism conferred through high expression levels of βIII tubulin, the lack of difference in IC_50_ between MES-SA/Dx5 and MES-SA is reasonable. However, when the taxane analogs were co-administered with verapamil to these cell lines it resulted in a decrease of IC_50_ values of MES-SA/Dx5 cells to levels that are similar to that of MES-SA cells, indicating a complete or near complete inhibition of P-gp. MES-SA cells treated with verapamil and either paclitaxel, Tx-D, or Tx-F have a statistically significant drop in IC_50_ values as compared to the same cells without verapamil. Since MES-SA cells do not express P-gp, there were likely off-target interactions occurring that contributed to the slight increase in cytotoxicity of these three compounds in combination with verapamil (Table 2) or that the P-gp expression was not detectable by western blot under the experimental conditions. Prolonged growth or incubation times of up to 48 or 72 hours may be needed to observe the induction and concomitant detection of P-gp expression by western blot.

K-562 (P-gp^−^) and K-562/R7 (P-gp^+^) cells were also treated with paclitaxel or analogs in combination with verapamil. The combination with verapamil did not result in a complete inhibition of activity, possibly due to the overwhelming expression of P-gp within the K-562/R7 cells. Paclitaxel plus verapamil resulted in about an 11-fold decrease in IC_50_ for K-562/R7 cells (from 440 nM to 41 nM), but the IC_50_ was still about an 8-fold greater than the IC_50_ of parental K-562 cells under the same conditions (5 nM). This was also seen for K-562/R7 cells treated with Tx-A plus verapamil, where there was an approximately 12-fold decrease in IC_50_ (from 266 nM to 22 nM), but it was still about 4-fold greater than K-562 under the same conditions (6 nM) (Table 2).

Moreover, combination treatments involving Tx-C, Tx-D or Tx-F with verapamil resulted in decreases in IC_50_ not only for K-562/R7 cells, but also for K-562 cells. As expected, there was a shift to a lower IC_50_ in K-562/R7 cells (which are P-gp^+^) to levels similar to that of K-562. This indicates that the presence of verapamil is almost completely inhibiting the mechanisms used by the cells to resist Tx-C, Tx-D or Tx-F treatments. However, the significant decrease in IC_50_ of parental K-562 cells treated with verapamil was an unexpected result. For each of Tx-C, Tx-D and Tx-F the decrease in IC_50_ for K-562 with and without verapamil treatment was greater in proportion than the decrease in IC_50_ for K-562/R7 cells treated with verapamil (Table 2).

### Computational Simulations

#### Binding to the Taxane Binding Site

Complexes of β-tubulin with a bound taxane, including paclitaxel, docetaxel and the seven paclitaxel derivatives (Tx-A, Tx-B, Tx-C, Tx-D, Tx-E, Tx-F, and Tx-G) were generated through *in silico* modeling. The binding site considered was the main taxane site on β-tubulin [1]. The complexes were simulated using molecular dynamics computations for up to 11 ns until root-mean-square-deviation (RMSD) equilibration was obtained. Based on the stabilization observed in the last few nanoseconds of the simulation, the final 2 ns were judged to be equilibrated and were used for both Molecular Mechanics/Poisson–Boltzmann Surface Area (MM/PBSA) and Molecular Mechanics/Generalized Born Surface Area (MM/GBSA) calculations, as well as for normal mode analysis. Table 3 lists the binding energies, Δ*G*_*b*_, obtained from both the MM/PBSA and MM/GBSA calculations, as well as experimental binding energies obtained from the literature for paclitaxel and docetaxel. As the table shows, considering relative binding energies only, docetaxel ranks first and slightly better than paclitaxel for both the MM/PBSA and MM/GBSA values, which agrees with the experimental measurements and supports the validity of the computational model used. The predicted binding energies show that paclitaxel is a stronger binder than all the taxane derivatives. The calculations also show that Tx-D and Tx-F are predicted to bind more strongly than Tx-A and Tx-C for both the MM/PBSA and MM/GBSA values.

**Table 3:** Results of the MM/PBSA and MM/GBSA Calculations.

Molecule	Δ*G*_*b*_ (MM/GBSA)	Δ*G*_*b*_ (MM/PBSA)	Δ*G*_*b*_ (Exp.)*
Paclitaxel	−28.3±1.1	−18.6±1.1	−10.1±0.01
Docetaxel	−32.6±1.0	−19.8±1.0	−10.7±0.05
Tx-A	−23.3±0.9	−10.9±0.9	N/D^†^
Tx-B	−10.4±1.0	−3.2±1.0	N/D^†^
Tx-C	−19.5±0.9	−8.7±1.0	N/D^†^
Tx-D	−25.5±1.0	−16.9±1.1	N/D^†^
Tx-E	−13.4±1.0	4.9±1.1	N/D^†^
Tx-F	−25.9±1.0	−18.1±1.0	N/D^†^
Tx-G	−28.2±1.2	−18.4±1.1	N/D^†^

Energies listed include the entropy calculated through normal mode analysis. More negative values indicate stronger binding. Energies are in kcal/mol at a temperature of 300 K. All computed energies are expressed as mean ± standard error.

* Experimental energies from Buey et al. [63] are given for comparison.

^†^ N/D: no data.

**Fig 7:**
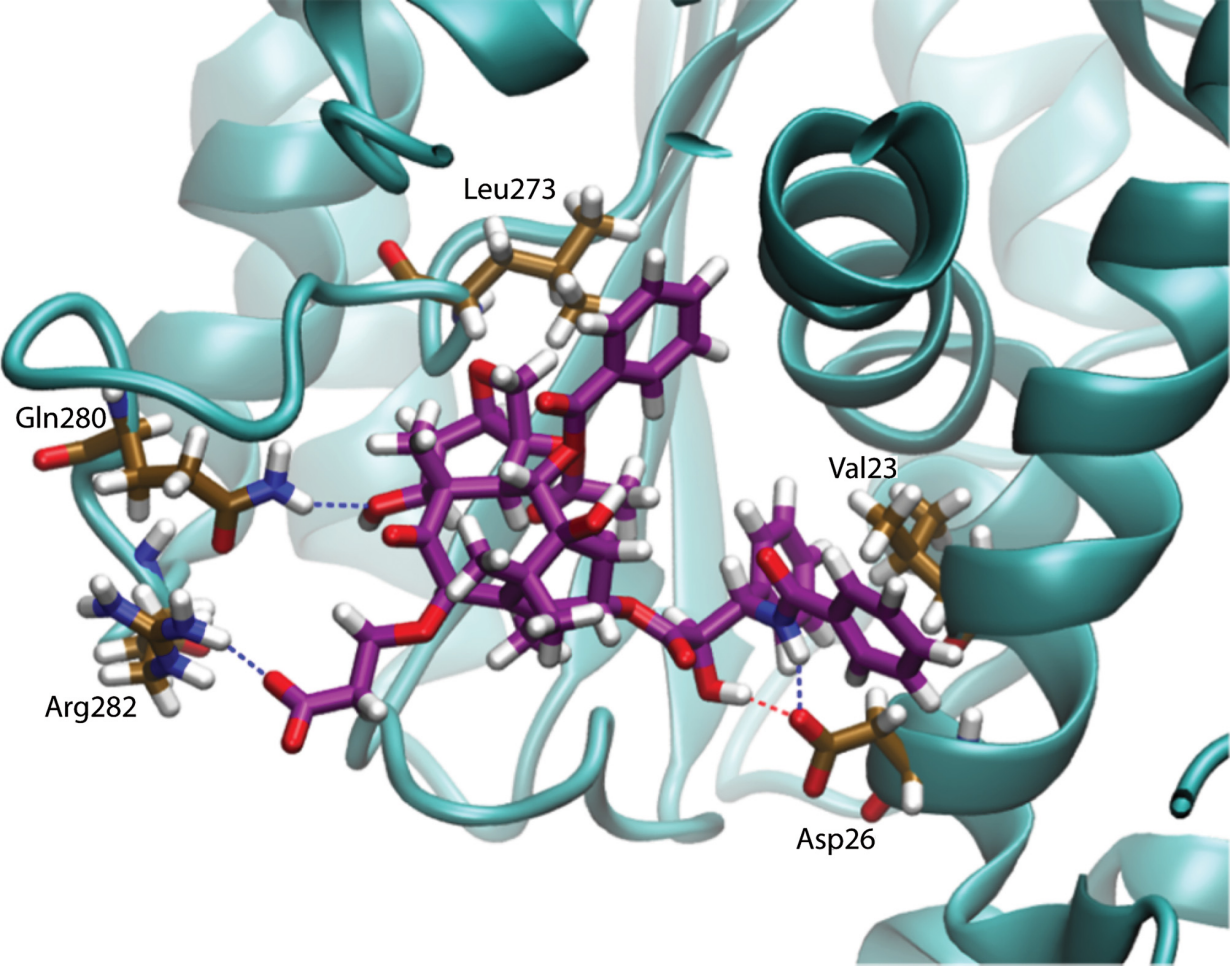
Binding Mode of Tx-F in the Taxane Binding Site at the End of Molecular Dynamics Simulation. The tubulin structure is shown as a cyan cartoon. Important residues are depicted in gold and the Tx-F molecule is purple (oxygen atoms are red, nitrogen atoms are blue, and hydrogen atoms are white). Dashed lines represent hydrogen bonds. Tx-F forms a very strong salt bridge with Arg284 of the M-loop of β-tubulin.

Upon analysis of the binding modes of Tx-D and Tx-F it was observed that they form very strong salt bridges with charged arginine residues of the M-loop of β-tubulin (Fig 7). These salt bridges are absent in the binding modes of the other derivatives and they are responsible for the strong binding energies of Tx-D and Tx-F. In fact, the enthalpy of Tx-F binding is even greater than that of paclitaxel. However, the freezing of the M-loop induced by the Tx-F salt bridge raises the entropic loss upon its binding, and gives an overall binding energy of Tx-F that is weaker than paclitaxel.

#### Binding to the Intermediate Nanopore Site

A model of the intermediate paclitaxel binding site [2] was used to dock paclitaxel and four of the taxane derivatives. Scoring of the structures using four different docking protocols is provided (Table 4). The docking protocols varied in the size of the binding site and whether the receptor was flexible (induced fit protocol) or rigid:

1. Induced fit protocol. Large binding site (residues within 9 Å of paclitaxel in Fig 2).
2. Induced fit protocol. Small binding site (residues within 4.5 Å of paclitaxel in Fig 2).
3. Induced fit protocol. Ligand defines binding site (residues in contact with paclitaxel in Fig 2).
4. Rigid receptor. Ligand defines binding site (residues in contact with paclitaxel in Fig 2).

**Table 4:** Docking Scores for Docking to the Intermediate Binding Site Using Four Different Protocols.

	Protocol 1: Induced fit, large site	Protocol 2: Induced fit, small site	Protocol 3: Induced fit, ligand site	Protocol 4: Rigid receptor, ligand site
Paclitaxel	−9.4	−9.2	−9.2	−9.5
Tx-A	−9.8	−9.4	−9.5	−7.8
Tx-C	−9.0	−8.1	−8.3	−7.5
Tx-D	−9.8	−8.7	−8.7	−8.5
Tx-F	−9.2	−8.4	−9.0	−8.9

Units are kcal/mol.

The conformations of the structures generated using the first three protocols, which allow for receptor flexibility, tended to result in migration of the taxanes away from the intermediate binding site (and also away from the site identified by Maccari et al. [48]) and towards the β_4_ M-loop. Therefore, in order to limit this migration, the results from the fourth protocol, using a rigid receptor, are presented (Table 4). It is worth noting that the use of an induced fit procedure may permit exploration of the potential energy surface of the protein-ligand complex by following the pathway through which the drug moves away from the intermediate binding site and towards the luminal binding site via interactions with the M-loop.

The rigid receptor results show that paclitaxel has the strongest binding, followed by Tx-D and Tx-F, with Tx-A and Tx-C having the weakest binding. This trend for the rigid docking affinities in the intermediate site is the same as was observed for the calculated affinities at the luminal site.

The computational method used to calculate the binding energies of the derivatives to the main taxane site, molecular dynamics with MM/PBSA and MM/GBSA, is relatively accurate. By contrast the method used to calculate the binding for the intermediate nanopore site, molecular docking, is of lower accuracy but requires considerably less computational time. The binding energy values calculated by these two methods are not expected to be directly comparable; that is why we have only tried to compare the rank orders of the compounds. Another limitation of both methods is that they were only used to study binding to specific sites that we had already identified; neither method was capable of providing any information about other potential higher affinity binding sites. Finally, neither method was designed to study the details of traversal of a taxane molecule through the nanopore to the final binding site.

#### Permeability

Since binding energy is not enough in predicting the potency of a drug, we also tried to determine if the permeability of the drug across cell membranes could potentially explain the findings from paclitaxel or its analogs; to this end, we have used human effective jejunal permeability (S+Peff) using ADMET predictor software (ADMET Predictor, Simulations Plus, Lancaster, CA, USA). Based on the predicted permeability of each drug as well as its predicted binding energy, we calculated a scoring function that can be used in ranking the potency of each drug. The scoring function was as follows:

$${\text{Score}}_{i}=\frac{\Delta G_{i}}{\Delta G_{\text{min}}}+\frac{P_{i}}{P_{\text{min}}},$$ (3)

where the index *i* specifies the ligand studied, min specifies the lowest value among all the studied ligands and *P* is the predicted permeability. Dividing by the lowest values was used to standardize the two quantities (Δ*G* and *P*) and allow for their addition.

Table 5 lists the results obtained from this analysis as well as predicted permeabilities and experimental binding energies for positive controls.

**Table 5:** Score combining binding energy and permeability.

	Δ*G*_*b*_ *	S+Peff^†^	Score^‡^	IC_50_ ^§^
Paclitaxel	−28.3	0.12	3.4	6
Tx-A	−23.3	0.23	5.4	11
Tx-C	−19.5	0.23	5.3	16
Tx-D	−25.5	0.06	2.1	96
Tx-F	−25.9	0.05	1.9	68

* Δ*G*_*b*_ are energy values in kcal/mol using the MM/GBSA method (Table 3).

^†^ S+Peff is the human jejunal effective permeability expressed in 10^4^ cm/s.

^‡^ The score is calculated based on a function combining the effect of binding energy and permeability.

^§^ IC_50_ is the average IC_50_ in nM for the breast cancer cell lines (Table 1).

## Conclusions

This study examined a set of novel paclitaxel derivatives that were specifically designed to overcome the drug resistance associated with residue changes between β-tubulin isotypes in a putative intermediate binding site. The main idea was to modify functional groups to restore a hydrogen bond lost due to the replacement of Ser275 with Ala275 in βIII tubulin. We synthesized four different compounds that were predicted to probe both increased and decreased affinity for the βIII tubulin structure. This premise was then tested experimentally on both purified tubulin isotypes and in cancer cell lines. The results did not support the predictions that the proposed residues would impact tubulin polymerization and therefore the cytotoxic potential. Instead, we observed that all four paclitaxel analogs synthesized were less potent than paclitaxel. We then proceeded to molecular modeling of the interactions in order to understand the experimental data. We conclude that neither binding to the intermediate binding site nor the final binding site is sufficient to explain the activity of the taxanes studied. This conclusion can be of great value when designing the next generation of paclitaxel derivatives and analogs for cancer chemotherapy applications.

The results are also consistent with recent analyses concerning the parent compound, paclitaxel. The mechanism by which paclitaxel stabilizes microtubules has been the subject of several studies, which demonstrated that upon binding the compound displaces residues in the M-loop and it was proposed that this moves the M-loop towards the adjacent tubulin unit in the microtubule to stabilize microtubules by enhancing lateral contacts, particularly with the β:H1–S2 loop, β:H3, and the β:H2–S3 loop [47,50,51]. Paclitaxel is also believed to impart microtubule stability by allosterically reversing the conformational changes to αβ-tubulin that occur upon the hydrolysis of GTP to GDP in β-tubulin [51,52]. The conformation of the αβ-tubulin heterodimer, with either GTP or GDP bound to β-tubulin, was examined [53–60] with few studies considering the effects of taxanes [51,52,61]. Local and global structural changes that occur within αβ-tubulin upon paclitaxel binding to microtubules were also identified [57,61]. Hydrogen-deuterium exchange (HDX) detected structural changes in both the α- and β-tubulin subunits [61]. Most recently, high-resolution microtubule structures have indicated that lateral contacts play a passive role in microtubule stability and are not substantially affected by paclitaxel binding, which instead stabilizes microtubules by increasing longitudinal contacts and easing conformational strain [52].

Limitations of the study design potentially includes the limited availability of the synthetic analogs due to synthesis constraints and their relative purity, as well as the limited choice of cell lines and the restrictive experimental conditions (analysis at 24 hours). However, the overall strength of the study lies in the multitude of analysis, viz., western blot for expression of protein markers known to mediate drug resistance, polymerization assays, assays using cell line models, and the broad selection of cell lines (breast cancer, non-breast cancer, adherent and suspension cultures). The results and interpretations are therefore independent of the cell lines used and the emerging theme that the mechanisms of resistance are far more complex than anticipated than can be explained from the putative interactions of specific moieties between drug and β-tubulin and warrant further studies. Rational drug design and its utility in the clinic depend on improvements in computational approaches and refinements in prediction algorithms.

An insight gained from these results is the importance of an iterative approach to drug design. Computational results alone should not be the foundation for engineering new taxane derivatives. Rather, the biological activity in cell line models should be used to refine the computational models, which can in turn provide more understanding regarding the mechanism of the drugs.

A future step planned is to investigate the effect of single-residue mutations tubulin on the activity of taxanes. This would be an excellent way to test the prediction that specific residues in the microtubule nanopore are important for taxane binding, particularly β-tubulin Ser275 and Ser278. To enable such a study for taxanes and other tubulin-binding compounds we have developed a recombinant tubulin expression system, which we have recently used to study colchicine derivatives [62].
